# Plasma Proteins Associated With Incident Acute Myocardial Infarction in Asians With Type 2 Diabetes

**DOI:** 10.1016/j.jacasi.2026.06.033

**Published:** 2026-08-12

**Authors:** Resham Lal Gurung, Huili Zheng, Jia Le Ivan Tan, Hao Wu, Sylvia Liu, Rajkumar Dorajoo, Jian-Jun Liu, Su Chi Lim

**Affiliations:** a Clinical Research Unit, Khoo Teck Puat Hospital, Singapore; b Cardiovascular and Metabolic Disorders Signature Research Program, Duke-NUS Medical School, Singapore; c Genome Institute of Singapore, A∗STAR, Singapore; d Department of Paediatrics, Yong Loo Lin School of Medicine, National University of Singapore, Singapore; e Saw Swee Hock School of Public Health, National University of Singapore, Singapore; f Lee Kong Chian School of Medicine, Nanyang Technological University, Singapore

**Keywords:** acute myocardial infarction, biomarkers, proteomics, type 2 diabetes

## Abstract

**Background:**

Individuals with type 2 diabetes (T2D) have increased risk of acute myocardial infarction (AMI). Cardiovascular risk within T2D is heterogeneous and not fully explained by conventional risk factors, highlighting the need for improved biological risk stratification.

**Objectives:**

The authors aimed to identify circulating proteins associated with incident AMI in T2D, evaluate their incremental predictive value, and explore potential causal relationships.

**Methods:**

In 1,830 adults with T2D from the prospective SMART2D cohort, 1,457 plasma proteins were quantified at baseline using Olink proximity extension assays. Participants were followed for incident AMI through linkage with the Singapore Myocardial Infarction Registry over a median of 10.2 years (IQR: 9.1-10.7). Multivariable Cox regression assessed protein-AMI association. Incremental predictive performance was evaluated using Harrell's C-statistic, likelihood ratio tests (LRTs), net reclassification index (NRI), and integrated discrimination index (IDI). Two-sample Mendelian randomization, using cis-protein qualitative-trait loci from SMART2D and outcome data from Biobank Japan (14,992 cases; 146,214 controls), examined causal associations.

**Results:**

During follow-up, 223 participants (12.2%) developed AMI. Seventeen proteins were independently associated with AMI after Bonferroni correction (*P* < 3.43 × 10^−5^), with the strongest associations observed for NEFL (HR: 1.70, 95% CI: 1.43-2.01), EDA2R (HR: 2.25, 95% CI: 1.69-2.97), and CHRDL1 (HR: 2.69, 95% CI: 1.83-3.94). An 8-protein panel improved risk classification beyond clinical variables (LRT *P* = 0.002; IDI: 0.049, 95% CI: 0.022-0.128, *P* = 0.002; and NRI: 0.318, 95% CI: 0.112-0.411, *P* = 0.008). Genetically predicted plasma FGF5 levels were associated with myocardial infarction risk.

**Conclusions:**

Plasma proteomics improves AMI risk stratification in T2D, and FGF5 may play a role in cardiovascular risk in Asian populations.

Cardiovascular diseases remain the leading cause of mortality worldwide.^1^ Acute myocardial infarction (AMI) is a major contributor to cardiovascular death, and individuals with diabetes have up to 4-fold higher risk of AMI compared with those without diabetes,^2^, ^3^, ^4^ along with increased recurrent cardiovascular events and mortality.^5^ This burden is particularly pronounced in Asian populations, who demonstrate heightened susceptibility to type 2 diabetes (T2D) and earlier onset of coronary artery disease compared with Western populations, suggesting population-specific biological mechanisms.^6^^,^^7^

In Singapore, the incidence of AMI is projected to increase substantially over the coming decades, with estimates suggesting a nearly 2-fold rise between 2025 and 2050.^8^ Despite advances in preventive therapies, individuals with T2D exhibit marked heterogeneity in AMI risk that is not fully captured by conventional risk factors. This residual risk highlights the need for improved risk stratification strategies that reflect the complex biological processes underlying atherothrombosis in diabetes.

Circulating plasma proteins represent functional intermediates linking genetic predisposition and environmental exposures.^9^^,^^10^ Unlike traditional risk factors, proteomic biomarkers can integrate signals across multiple biological pathways involved in inflammation, endothelial dysfunction, and metabolic stress, processes central to diabetic cardiovascular diseases.^9^^,^^11^, ^12^, ^13^ Comprehensive proteomics profiling offers an opportunity to identify novel molecular signatures associated with AMI and improve the biological characterization of cardiovascular risk in T2D. Most prior studies have examined selected protein panels, mixed populations with and without diabetes, or predominantly Western cohorts.^14^^,^^15^ However, current knowledge on the association of plasma proteins and incident AMI in Asian populations with T2D remains limited. Therefore, in this prospective study, we evaluated 1,457 plasma proteins in Asians with T2D to identify those associated with incident AMI and explored the potential causal role using Mendelian randomization approaches.

## Methods

### Study population

SMART2D is a prospective cohort study designed to identify clinical and molecular determinants of vascular and nonvascular complications in Southeast Asian patients with T2D.^16^ Between August 2011 and March 2014, a total of 2,057 adults with physician-diagnosed T2D were consecutively recruited from a regional hospital and an adjacent primary care facility in Singapore. T2D was diagnosed according to prevailing American Diabetes Association criteria after exclusion of type 1 diabetes mellitus and diabetes attributable to specific causes.^17^ Individuals with pregnancy-related diabetes with manifest infection, autoimmune disease, or cancer on active treatment were excluded from cohort enrollment.^16^^,^^18^ Among 1,932 participants with complete baseline data, 67 individuals with baseline estimated glomerular filtration rate (eGFR) <30 mL/min/1.73 m^2^ and 35 with prior AMI were excluded, leaving 1,830 participants for analysis (Supplemental Figure 1). This study was approved by the Singapore National Healthcare Group Domain Specific Review Board, and all participants provided written informed consent.

### Clinical outcome ascertainment

The primary endpoint was incident AMI. Events were identified through data linkage with the Singapore Myocardial Infarction Registry, which captures all myocardial infarction (MI) cases diagnosed by certified physicians meeting at least 2 of the following criteria: 1) symptoms of AMI; 2) elevated cardiac biomarkers; or 3) electrocardiographic abnormalities consistent with AMI. Time-to-event was calculated from enrollment to first AMI, death, or censoring (December 31, 2022), whichever occurred first.

### Proteomic profiling

Details of SMART2D plasma proteomic sampling have been described previously.^19^ Fasting plasma samples were analyzed using the Olink Explore 1536 proximity extension assay. Samples were flagged if the internal controls deviated >0.3 normalized protein expression (NPX) units from the plate median. Fifteen proteins with >80% failed quality control were excluded, leaving 1,457 proteins (1,448 unique) for analysis. NPX values represent log_2_-transformed relative protein concentrations. Values below the detection limit were retained. Intra-assay coefficient of variation ranged from 7% to 8%, and interassay variation ranged from 11% to 14%.

### Covariates

Ethnicity, smoking status, diabetes duration, and cardiovascular history were obtained via questionnaire and verified through electronic medical records. Medication data were obtained from dispensing records. No participants used sodium-glucose cotransporter 2 (SGLT2) inhibitors at baseline, while only 4 participants used glucagon-like peptide-1 receptor agonists (GLP-1RA) at baseline. Blood pressure was measured 3 times by a semiautomated blood pressure monitor, and the mean value was used. Hemoglobin A1c was quantified by a point-of-care immunoassay analyzer (DCA Vantage Analyzer, Siemens). Serum high-density lipoprotein cholesterol, low-density lipoprotein cholesterol, triacylglycerol, and creatinine were quantified by enzymatic methods (Cobas Integra 700; Roche Diagnostics). The eGFR was estimated based on serum creatinine by the 2009 Chronic Kidney Disease–Epidemiology Collaboration formula.^20^ Urinary albumin was measured by an immunoturbidimetric assay (Cobas, Roche Diagnostics), and albuminuria was expressed as urine albumin-to-creatinine ratio (uACR). Plasma specimens were collected after overnight fasting, aliquoted, and stored at −80 °C.

### Statistical analysis

Baseline characteristics were compared between participants with and without incident AMI using the Wilcoxon rank-sum test for continuous variables and chi-square tests for categorical variables. The number of participants with complete data for each clinical and biochemical variable is reported in Table 1. Missing data (<1%) could not be assumed to be missing completely at random (MCAR) and were handled by listwise deletion.

**Table 1:** Baseline Characteristics by AMI Events During Follow-Up

	n	Overall (N = 1,830)	No AMI (n = 1,607)	AMI (n = 223)	*P* Value
Index age, y	1,830	57 (51-64)	57 (51-64)	59 (53-66)	0.013
Male	1,830	921 (50.3)	786 (48.9)	135 (60.5)	0.001
Ethnicity	1,830				0.023
Chinese		950 (51.9)	854 (53.1)	96 (43.0)	
Malay		368 (20.1)	311 (19.3)	57 (25.6)	
Indian		442 (24.1)	384 (23.9)	58 (26.0)	
Other		70 (3.8)	58 (3.6)	12 (5.4)	
CVD history	1,829	116 (6.3)	90 (5.6)	26 (11.7)	<0.001
Smoking status	1,827				<0.001
Current smoker		158 (8.6)	124 (7.7)	34 (15.4)	
Ex-smoker		130 (7.1)	109 (6.8)	21 (9.5)	
Never smoked		1,539 (84.2)	1,373 (85.5)	166 (75.1)	
BMI, kg/m^2^	1,827	26.9 (24.1-30.6)	26.9 (24.1-30.6)	27.1 (24.3-30.2)	0.726
Diabetes duration, y	1,829	10 (4-15)	9 (4-15)	11 (6-20)	<0.001
HbA1c, %	1,830	7.5 (6.8-8.5)	7.4 (6.8-8.4)	8.2 (7.0-9.3)	<0.001
HbA1c, mmol/mol	1,830	58 (51-69)	57 (51-68)	66 (53-78)	
MAP, mm Hg	1,830	99 (92-106)	98 (92-105)	100 (94-107)	0.005
SBP, mm Hg	1,830	138 (127-151)	137 (126-150)	144 (131-157)	<0.001
DBP, mm Hg	1,830	79 (73-85)	79 (73-85)	79 (72-86)	0.710
HDL-C, mmol/L	1,829	1.25 (1.06-1.49)	1.26 (1.07-1.50)	1.19 (0.98-1.43)	0.001
LDL-C, mmol/L	1,829	2.61 (2.20-3.20)	2.61 (2.20-3.17)	2.65 (2.17-3.36)	0.199
TG, mmol/L	1,829	1.39 (1.03-1.90)	1.38 (1.03-1.89)	1.56 (1.08-2.04)	0.018
eGFR, mL/min/1.73 m^2^	1,830	94 (74-105)	95 (77-106)	79 (56-96)	<0.001
uACR, mg/g	1,819	21 (6-83)	19 (6-68)	57 (14-307)	<0.001
Lipid-lowering agent	1,830	1,498 (81.9)	1,299 (80.8)	199 (89.2)	0.002
RAS antagonist	1,830	1,072 (58.6)	912 (56.7)	160 (71.7)	<0.001
Insulin	1,830	484 (26.6)	389 (24.3)	95 (43.0)	<0.001

Values are presented as median (IQR) or n (%).

AMI = acute myocardial infarction; BMI = body mass index; CVD = cardiovascular disease; DBP = diastolic blood pressure; eGFR = estimated glomerular filtration rate; HbA1c = glycated hemoglobin; HDL-C = high-density lipoprotein cholesterol; LDL-C = low-density lipoprotein cholesterol; MAP = mean arterial pressure; RAS = renin-angiotensin system; SBP = systolic blood pressure; TG = triacylglycerol; uACR = urine albumin-to-creatinine ratio.

Cox proportional hazards regression models were used to evaluate associations between each protein (per 1-unit NPX increase) and incident AMI. The proportional hazards assumption was tested using the Schoenfeld residuals and was not violated any proteins (false discovery rate [FDR] >0.05). Covariates included in the multivariable Cox models were measured at baseline and selected a priori: age, sex, ethnicity (Chinese as reference), active smoking (yes or no), cardiovascular disease history (yes or no), body mass index (BMI), diabetes duration, hemoglobin A1c, mean arterial pressure, high-density lipoprotein cholesterol, low-density lipoprotein cholesterol, log-transformed triacylglycerol, lipid-lowering medication (yes or no), eGFR, and log-transformed uACR. Multiple testing correction was applied across the 1,457 analyzed proteins using the Bonferroni and Benjamini-Hochberg procedures, where *P* < 0.05/1457 = 3.43 × 10^−5^ and FDR <0.05, respectively, were considered statistically significant.

Proteins associated with incident AMI in the multivariable Cox model (Bonferroni-corrected *P* < 3.43 × 10^−5^) were taken forward for sensitivity and subgroup analyses. Sensitivity analyses included Fine-Gray competing-risk regression and an analysis restricted to nonfatal AMI as outcome. Non-AMI deaths were treated as the competing event, and subdistribution hazard ratios were reported. Nonfatal AMI was defined as AMI not resulting in death within 1 month.

To explore whether protein-AMI associations differed across subgroups, analyses were performed stratified by sex (male or female), baseline cardiovascular disease history (yes or no), baseline kidney function (eGFR ≥60 mL/min/1.73 m^2^ and uACR <30 mg/g, or otherwise), and age group (by tertiles). These analyses were prespecified based on known biological and clinical heterogeneity in AMI risk. Effect modification was assessed through multiplicative interaction analysis, whereby a product term between each protein and the subgroup covariate of interest was included alongside the respective main effects in Cox proportional hazards regression models fitted to the entire cohort. A statistically significant interaction term (*P* < 0.05) was taken to indicate effect modification.

### Replication in the UK Biobank cohort

To assess the generalizability of our findings, the associations of the 17 identified proteins with incident AMI were examined in the UK Biobank cohort. Published hazard ratios with 95% confidence intervals were from Ho et al,^9^ in which associations were adjusted for age, sex, race, deprivation index, education, smoking status, alcohol consumption, physical activity, dietary quality, BMI, systolic blood pressure, and diabetes. As reported in the original publication, *P* values were corrected for multiple testing using the Bonferroni procedure.

### Pathway enrichment analysis

Proteins associated with incident AMI in the multivariable Cox model (FDR <0.05) were used for pathway enrichment analysis using g:Profiler.^21^ The background gene set comprised the 1,457 proteins measured in this study, with at least one annotation in the Ensembl database of *Homosapien* (human). Multiple testing was corrected using the Benjamini-Hochberg procedure, with statistical significance set at FDR <0.05. Enrichment analysis was restricted to the Gene Ontology (GO) domains of biological process (GO: BP), molecular function (GO:MF) and cellular component (GO:CC) as well as the Kyoto Encyclopedia of Genes and Genomes (KEGG) pathways.

### Prediction analysis

Data were randomly split into a training set (n = 1,270 [70%], 12% with incident AMI) for protein identification and a test set (n = 560 [30%], 12% with incident AMI) for model comparison. As our aim was to evaluate the incremental value of specific proteins over clinical variables (variable utility) rather than generalizability of developed models to other populations (model transportability), models were refitted in the test set using proteins selected from the training sets. From the proteins associated with AMI in the multivariable Cox model after Bonferroni correction (*P* < 3.43 × 10^−5^), elastic net Cox regression, with clinical covariates retained a priori, was applied to identify a subset of proteins collectively predictive of AMI. The regularization parameter lambda was tuned via 10-fold cross-validation, and the mixing parameter alpha was selected through a grid search over values from 0.1 to 0.9 in increments of 0.1. Model discrimination was assessed using Harrell's C-statistic. Incremental improvement in model performance was evaluated by comparing models incorporating protein signatures against those based on traditional clinical variables alone in multivariable Cox model, the PREVENT model (age, sex, current smoking, eGFR, BMI, systolic blood pressure, high-density lipoprotein cholesterol, total cholesterol, antihypertensive medications, and lipid-lowering medications),^22^ and the Framingham Risk Score (age, sex, current smoking, systolic blood pressure, high-density lipoprotein cholesterol, total cholesterol, and antihypertensive medications).^23^ Likelihood ratio tests (LRTs) were used to assess improvement in model fit. The net reclassification index (NRI), integrated discrimination index (IDI), and decision curve analysis were used to assess improvements in risk classification and clinical utility over a 10-year follow-up period.

### Two-sample Mendelian randomization analysis

Genotype data were available in SMART2D participants from previous studies.^24^^,^^25^ Genome-wide association analyses for selected proteins were performed within SMART2D using a generalized linear mixed model adjusting for age, sex, and population stratification (principal component 1-3). Genetic instruments were selected based on genome-wide significance (*P* < 1 × 10^−8^), and instrument strength (*F*-statistic), and were restricted to cis-acting variants (within 1 Mb of the gene encoding the protein of interest). As single-SNP instruments were used, linkage disequilibrium clumping and pruning were not applicable.

A 2-sample Mendelian randomization framework was used to assess the causal relationship between plasma proteins and MI pathogenesis. The validity of Mendelian randomization analysis relies on 3 key assumptions: 1) robust genetic instrument-exposure association; 2) no association between genetic instrument and confounder; and 3) the genetic instrument affects the outcome only through the exposure.^26^ The instrumental variable-exposure coefficient was derived from the current study, while instrumental variable-outcome estimates were obtained from an MI genome-wide association study in the BioBank Japan project^27^ (see the Supplemental Methods). MI in BioBank Japan was identified using the International Classification of Diseases, 10th Revision (ICD-10) codes. Causal effects were estimated using the Wald ratio test.^28^^,^^29^

All analyses were performed using SPSS version 27, R version 4.3.2, and Stata SE version 18. Two-tailed *P* values <0.05 were considered statistically significant unless otherwise specified.

## Results

### Associations between plasma proteins and risk of incident AMI

Over a median follow-up of 10.2 years (IQR: 9.1-10.7; 16,994 patient-years), 223 incident AMI events occurred. Participants who developed AMI were older, more likely to be male, and of Malay ethnicity, had a history of cardiovascular disease, were current smokers, had longer diabetes duration, higher hemoglobin A1c, higher mean arterial pressure, and higher triglycerides, and poorer renal function (Table 1).

Among 1,457 proteins analyzed, 17 proteins (NEFL, EDA2R, CHRDL1, FCER2, CXCL13, COL4A1, RELT, TNFRSF10B, NT-proBNP, TNFRSF4, FGF5, COL18A1, CCN5, JAM2, LAMA4, CD74, and IGFBP4) remained significantly associated with incident AMI after multivariable adjustment, including baseline kidney function (Bonferroni correction *P* < 3.43 × 10^−5^) (Figure 1, Supplemental Table 1). The most robust association were observed for neurofilament light chain (NEFL; HR: 1.70, 95% CI: 1.43-2.01, *P* = 6.52 × 10^−10^), Tumor necrosis factor receptor superfamily member 27, also known as EDA2R (TNFRSF27; HR: 2.25, 95% CI: 1.69-2.97, *P* = 1.41 × 10^−8^) and Chordin-like protein 1 (CHRDL1; HR: 2.69, 95% CI: 1.83-3.94, *P* = 4.35 × 10^−7^).

**Figure 1:**
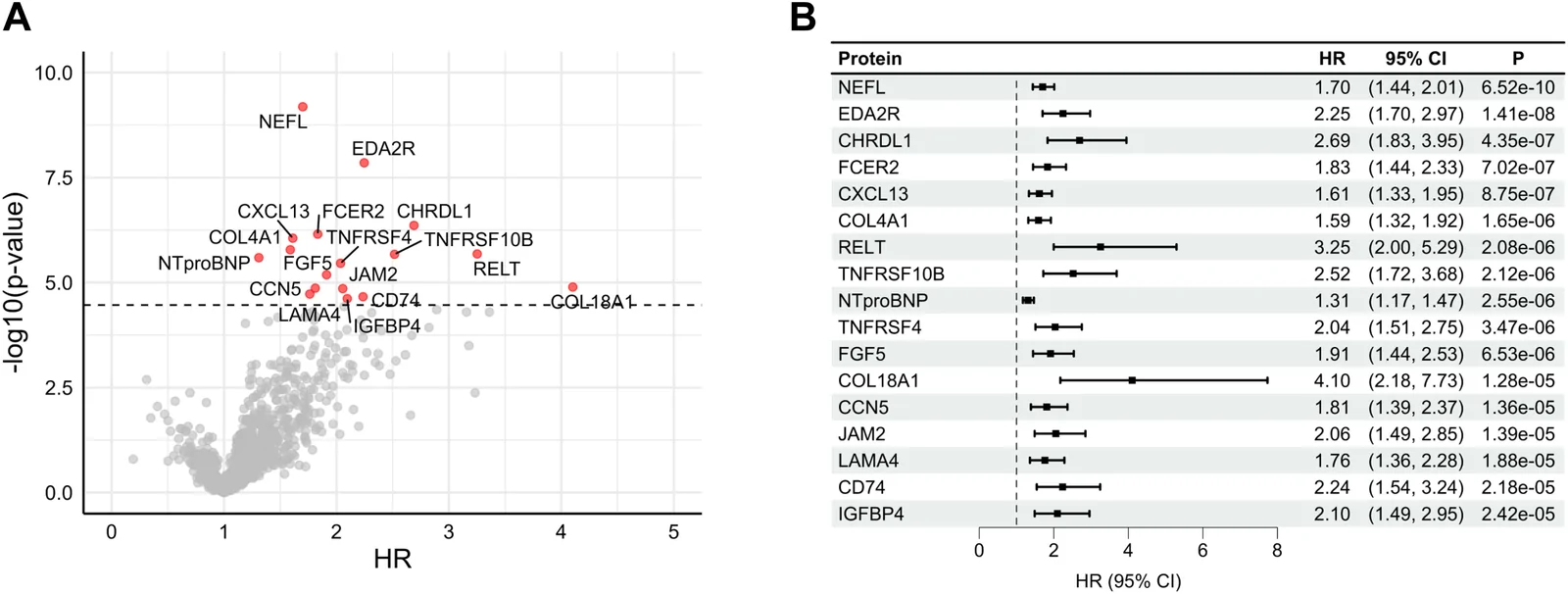
Associations Between Plasma Proteins and Risk of Incident AMI The model adjusted for age, sex, ethnicity, cardiovascular disease history, smoking status, body mass index, diabetes duration, hemoglobin A1c, mean arterial pressure, high-density lipoprotein cholesterol, low-density lipoprotein cholesterol, triacylglycerol, use of lipid-lowering medication, statin baseline estimated glomerular filtration ratio, and log-transformed urine albumin-to- creatinine ratio. (A) The red dot in the volcano plot indicates proteins (n = 17), with *P*_adj_ < 3.43 × 10^−5^. (B) The forest plot provides corresponding HR (95% CI).

### Sensitivity analysis of the proteins identified from primary analysis

From competing risk regression analysis that accounted for 59 non-AMI deaths, 8 proteins (EDA2R, FGF5, CHRDL1, NEFL, FCER2, COL4A1, CCN5, CXCL13) remained significantly associated with AMI (*P* < 3.43 × 10^−5^) (Supplemental Table 2). Additionally, 22 proteins were associated with nonfatal AMI independent of traditional risk factors, of which 14 proteins overlapped with total AMI events (Supplemental Table 3).

### Subgroup analyses

In exploratory subgroup analyses, we observed notable protein-AMI associations across clinically relevant patient characteristics (Supplemental Tables 4 to 7). FGF5 demonstrated sex-specific associations (*P*-interaction = 0.036), with higher HR and only significant in males (*P*_adj_ = 7.06 × 10^−5^). EDA2R (*P*-interaction = 0.048), IGFBP4 (*P*-interaction = 0.031), and NEFL (*P*-interaction = 0.007) interacted with diabetic kidney disease (DKD). Specifically, EDA2R demonstrated significant associations in patients with DKD (*P*_adj_ = 2.35 × 10^−6^), while NEFL's significance was observed only in non-DKD patients (*P*_adj_ = 9.50 × 10^−6^). Age-related differences were observed for LAMA4 (p-interaction = 0.037) and TNFRSF4 (*P*-interaction = 0.038), with LAMA4 showing significant association with AMI risk in older age group (*P*_adj_ = 3.06 × 10^−6^).

### Validation in independent cohorts

In an external validation using the UK Biobank cohort, the associations of 17 AMI-associated proteins identified in SMART2D showed directional consistency. Of these, 14 proteins were also statistically significant with incident MI in the UK Biobank cohort (Supplemental Table 8).

### Pathway enrichment

Among 1,457 proteins analyzed, 125 proteins remained significantly associated with incident AMI after multivariable adjustment including baseline kidney function, with FDR <0.05. Pathway enrichment analysis revealed that AMI-associated proteins were predominantly enriched in pathways related to extracellular matrix structural constituent, structural molecule activity, death receptor activity, and cytokine-cytokine receptor interactions, highlighting the importance of inflammatory and apoptotic processes in diabetic cardiovascular disease (Supplemental Figure 2).

### Prediction of incident AMI

The 17 AMI-associated proteins showed positive intercorrelations (Supplemental Figure 3) and were positively correlated with metabolic risk factors and poorer baseline renal function (Supplemental Figure 4). In the training set, elastic net Cox regression, accounting for metabolic risk factors and interprotein correlations, retained 8 proteins: LAMA4, NT-proBNP, NEFL, FGF5, FCER2, COL4A1, CXCL13, and EDA2R. In the test set, these 8 proteins collectively achieved Harrell's C-statistic of 0.772 (95% CI: 0.714-0.831) for AMI risk prediction, comparable to the model with clinical variables alone (0.779, 95% CI: 0.728-0.830) (Supplemental Figure 5). Integrating the 8 proteins with clinical variables showed a modest improvement in discrimination (ΔC-statistic: 0.030, 95% CI: –0.001 to 0.062, *P* = 0.056). Nonetheless, the addition of 8 proteins significantly improved model fit on the likelihood ratio test (*P* = 0.002), improved risk classification based on both IDI (0.049, 95% CI: 0.022-0.128, *P* = 0.002) and NRI (0.318, 95% CI: 0.112-0.411, *P* = 0.008), and the demonstrated higher net clinical benefit on decision curve analysis compared to the model without proteins over the threshold probabilities below 0.4.

The 8-protein model also demonstrated comparable discrimination to the PREVENT model (C-statistic: 0.749, 95% CI: 0.694-0.805) and the Framingham Risk Score (C-statistic: 0.707, 95% CI: 0.647-0.768). Importantly, the addition of the protein signature to both established risk scores yielded consistent improvements in discrimination, with a ΔC-statistic of 0.037 (95% CI: 0.00001-0.073, *P* = 0.049) when added to the PREVENT model and 0.078 (95% CI: 0.026-0.130, *P* = 0.003) when added to the Framingham Risk Score.

### Additional analysis

#### Genetically determined plasma protein and MI risk

Genome-wide association analyses in the SMART2D cohort identified genome-wide significant loci (*P* < 1 × 10^−8^) for 3 of the 17 AMI-associated proteins: NT-proBNP (rs6668659; β [SE] = 0.288 [0.046], *P* = 2.65 × 10^−10^), FGF5 (rs16998073; β [SE] = 0.268 [0.016], *P* = 3.08 × 10^−66^), and FCER2 (rs1990975; β [SE] = −0.235 [0.028], *P* = 3.12 × 10^−17^) (Table 2, Supplemental Figures 6 to 8). The cis-protein quantitative trait loci identified for NT-proBNP (rs6668659), FGF5 (rs16998073), and FCER2 (rs1990975) were in linkage disequilibrium with lead variants rs198379, rs12509595, and rs2277989, respectively, in the UK Biobank cohort.^30^ The FGF5 variant rs16998073 (T allele) was also significantly associated with MI in an independent Asian population from the Biobank Japan project (OR: 1.07, 95% CI: 1.04-1.10; *P* = 1.40 × 10^−6^; 14,992 cases and 146,214 controls) (Table 2). Two-sample Mendelian randomization analyses suggest a potential causal relationship between circulating FGF5 levels and myocardial infarction risk (Table 2).

**Table 2:** Association of Genetically Determined Plasma Proteins and Myocardial Infarction

Protein	SNP	TA	TAF	**β** (95% CI)^a^	*P* Value	**β** (95% CI) of MI per Allele^b^	*P* Value	MR Estimated of MI per Unit Increment in Plasma Protein, OR (95% CI)	*P* Value
NT-proBNP	rs6668659	G	0.270	0.288 (0.198-0.378)	2.65E-10	−0.044 (−0.083 to −0.005)	2.60E-02	—	—
FGF5	rs16998073	T	0.368	0.268 (0.236-0.299)	3.08E-66	0.070 (0.041-0.099)	1.40E-06	1.30 (1.16-1.45)	<1×10^−5^
FCER2	rs1990975	T	0.186	0.235 (0.180-0.290)	3.12E-17	0.005 (−0.032 to 0.042)	7.90E-01	—	—

MI = myocardial infarction; MR = Mendelian randomization; NT-proBNP = N-terminal pro–B-type natriuretic peptide; TA = test allele; TAF = test allele frequency.

a Size estimated (unit increment per allele) extracted from the current study, adjusted for age, sex, and principal components 1 to 3.

b Data extracted from Biobank Japan, with MI data (cases = 14,992, controls = 146,214). Mendelian randomization analysis was only performed for FGF5.

## Discussion

In these prospective multiethnic Asian cohorts with T2D, we identified 17 circulating proteins associated with incident AMI independently of traditional clinical factors, including baseline kidney function, over more than a decade of follow-up, as summarized in the Central Illustration. An 8-protein panel further improved risk discrimination beyond traditional variables, supporting the potential utility of proteomic profiling to refine cardiovascular risk assessment in diabetes.

**Central Illustration:**
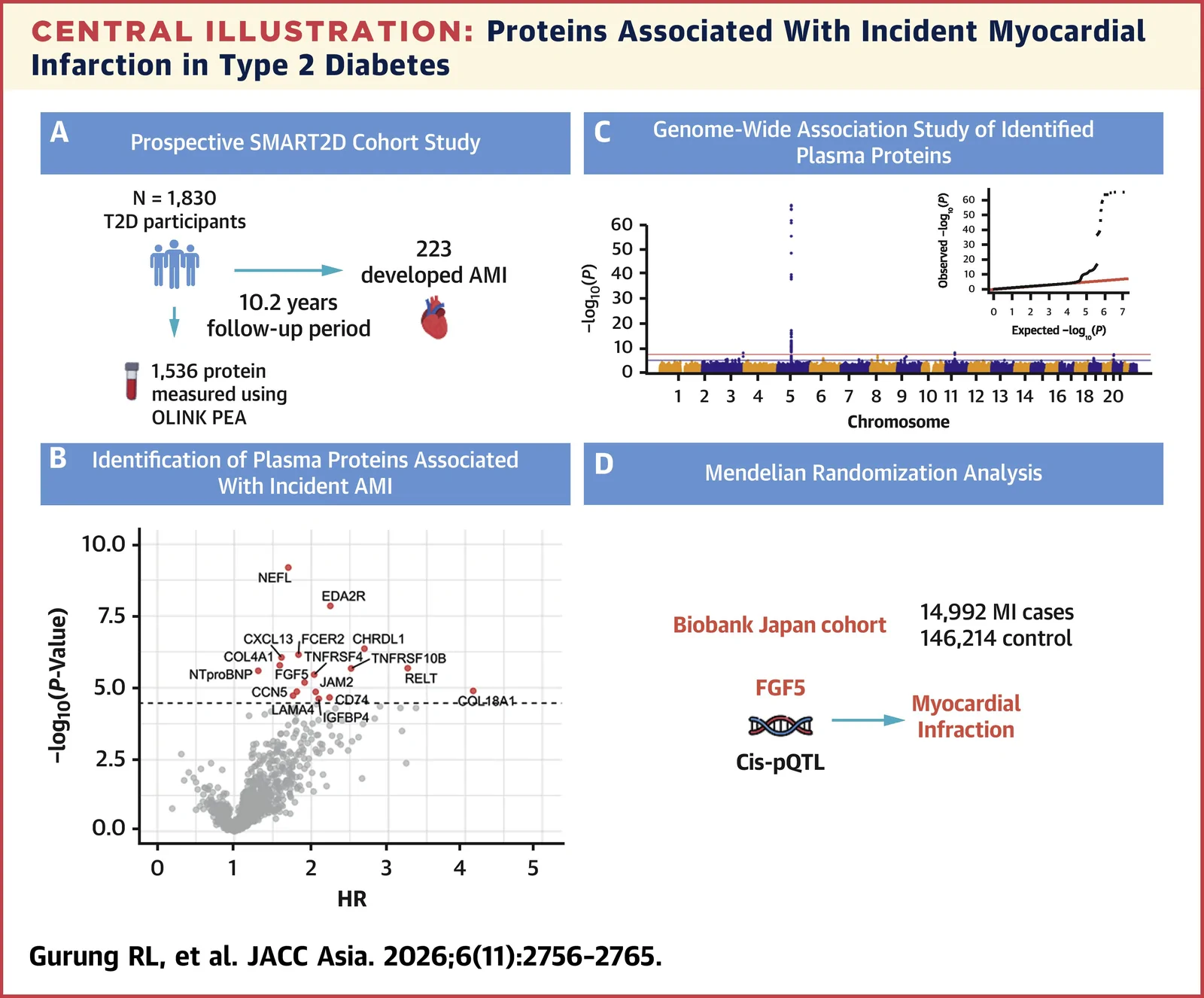
Proteins Associated With Incident Myocardial Infarction in Type 2 Diabetes (A) Prospective SMART2D cohort study. (B) Identification of plasma proteins associated with incident acute myocardial infarction (AMI). (C) Genome-wide association study of identified plasma proteins. (D) Mendelian randomization analysis to assess potential causal associations. MI = myocardial infarction.

All 17 proteins were positively associated with AMI risk. Several of these proteins showed significant correlations with traditional cardiovascular risk factors such as age, diabetes duration, blood pressure, and lipid levels. Notably, all 17 proteins were also significantly correlated with kidney function. Importantly, the associations between these proteins and incident AMI remained robust after adjustment for these traditional risk factors, indicating that the proteins may contribute to AMI risk through pathways not fully captured by conventional risk markers.

The strongest associations were observed for NEFL, EDA2R, and CHRDL1, implicating pathways related to neurovascular injury, inflammatory signaling, extracellular matrix remodeling, and apoptosis. In contrast to prior proteomic studies that examined mixed populations,^9^ smaller cohorts,^14^^,^^31^ or limited protein panels,^15^ our diabetes-focused design and broader proteomic coverage likely enabled the detection of T2D-specific cardiovascular signatures. To date, large-scale proteomic studies examining AMI risk in Asian populations with T2D remain limited, with most existing evidence derived from European cohorts comprising mixed populations of individuals with and without diabetes. Of the 17 proteins identified in our study, 14 have previously been associated with incident AMI in the UK Biobank cohort, of which 10 proteins (NT-proBNP, NEFL, EDA2R, CHRDL1, RELT, TNFRSF10B, COL18A1, CD74, TNFRSF4, and IGFBP4) have also been associated with coronary heart disease in Chinese populations through data from the China Kadoorie Biobank,^32^ lending cross-ethnic support to our findings. Importantly, 3 proteins (LAMA4, FGF5, and COL4A1) represent novel associations with incident AMI specifically in a T2D population. Collectively, our results extend existing literature by demonstrating the relevance of these proteomic biomarkers within an Asian T2D population, a group that has been substantially under-represented in large-scale cardiovascular proteomics research.

As summarized in Supplemental Table 9, the proteins identified in this study likely reflect distinct biological roles in relation to AMI risk. Some may have direct involvement in atherothrombotic pathways, including those implicated in inflammatory signaling (CXCL13, TNFRSF10B, TNFRSF4), extracellular matrix remodeling (COL4A1, COL18A1, LAMA4), endothelial integrity (JAM2), and vascular smooth muscle regulation (FGF5). Others more likely serve as markers of overall cardiovascular burden or systemic disease, including NT-proBNP (cardiac wall stress), NEFL (neuronal injury), IGFBP4 (metabolic dysfunction), and CD74 and FCER2 (broader inflammatory activation). A third group, including EDA2R, CHRDL1, RELT, and CCN5, may have mixed roles, contributing to disease pathways while also reflecting systemic disease burden.

An 8-protein panel (LAMA4, NT-proBNP, NEFL, FGF5, FCER2, COL4A1, CXCL13, and EDA2R) significantly improved AMI risk discrimination when added to established models, including the PREVENT and the Framingham Risk Score. Among these proteins, strong cis-protein quantitative trait loci were identified for NT-proBNP, FGF5, and FCER2. Mendelian randomization analyses supported a potential causal role for FGF5, a protein implicated in blood pressure regulation and vascular remodelling.^33^ The cis-pQTL identified in this study has also been associated with hypertension,^34^ strengthening biological plausibility. Exploratory subgroup analyses revealed that several protein-AMI associations were modified by clinically relevant characteristics, suggesting that cardiovascular risk in T2D may be driven by distinct biological pathways across patient subgroups. The sex-specific association of FGF5 and the DKD-dependent associations of EDA2R and NEFL highlight the heterogeneity of proteomic risk signatures within the diabetic population. These findings underscore the potential value of personalized, subgroup-specific biomarker panels in refining cardiovascular risk stratification in T2D.

### Study limitations

The strengths of this study include comprehensive proteomic profiling in a large, well-characterized cohort, long-term follow-up, and registry-based outcome ascertainment. However, several limitations merit consideration. First, residual confounding by unmeasured factors cannot be excluded, and proteins not included in the Olink panel may also contribute to cardiovascular risk. Although data missingness was low for most of the covariates included in this study, the data were not missing completely at random. Second, relative protein quantification rather than absolute protein concentrations was used. However, in a randomly selected subset of participants with T2D (n = 64), 5 proteins measures using both Olink and enzyme-linked immunosorbent assay demonstrated high concordance (median Spearman correlation, *r* = 0.83).^35^ Third, the proteins were measured at baseline, rather than longitudinally. In addition, we did not account for changes in risk factors in our analysis. Fourth, the observational study design precludes definitive causal inference. Although Mendelian randomization analyses provided supportive evidence for the potential causal role of selected proteins, these analyses were performed primarily in populations with and without diabetes owing to data availability, which may limit direct extrapolation to T2D-specific biology. Fifth, while protein selection was performed exclusively in the train set and remained fixed in the test set, the model coefficients were derived from the test set rather than applied from the train set. Hence, the performance metrics reflected locally optimized estimates rather than true out-of-sample prediction. This approach suffices to assess whether the identified proteins carry incremental utility over clinical variables but does not permit conclusions about the generalizability of model coefficients to other populations. External validation in an independent cohort, applying both the selected proteins and their derived coefficients without refitting the trained models, will be an important next step to confirm the transportability of these findings. Although the improvement in Harrell's C-statistic was modest with the addition of proteins to clinical variables, the consistent improvement in model fit, risk classification, and net clinical benefit supported the biological relevance of the identified protein. Sixth, as baseline information on antiplatelet therapy was not available for our cohort, we could not adjust for this potential confounder. Finally, the cohort comprised Southeast Asian individuals with T2D with minimal use of newer glucose-lowering therapies such as SGLT2 inhibitors or GLP-1 receptor agonists, and hence generalizability to other ethnic groups and the contemporary diabetes population requires confirmation.

## Conclusions

This large-scale proteomic analysis identified 17 circulating proteins associated with incident AMI in Asian individuals with T2D and demonstrated that an 8-protein panel improves risk discrimination beyond established clinical models. Genetic analyses further suggested a potential role for FGF5 in myocardial infarction risk. Together, these findings advance biological understanding of residual cardiovascular risk in T2D and provide a foundation for precision cardiovascular prevention strategies in Asian populations.

### Data Availability

Anonymous aggregated data may be available upon reasonable request from the corresponding authors and after approval from the Singapore National Healthcare Group Ethical Committee.

## Funding Support and Author Disclosures

This work was funded by Singapore National Medical Research Council Grants (MOH-001327-02, 001704-00, MOH-001688-00) and Khoo Teck Puat Hospital Science Translational and Applied Research grants (22206 and 21109). The funder had no role in the study design, data analysis, manuscript writing, or decision to submit for publication. The authors have reported that they have no relationships relevant to the contents of this paper to disclose.
