# Supplementary material for: Plasma Proteins Associated With Incident Acute Myocardial Infarction in Asians With Type 2 Diabetes
Source: JACC Asia. 2026 Aug 12;6(11):2756–65. doi: 10.1016/j.jacasi.2026.06.033 (PMC13750878; doi:10.1016/j.jacasi.2026.06.033)
Supplement: Supplementary file 1 [file mmc1.docx]

**Supplemental Material**

**Supplemental information.**

**Methods**

**Cohort description**

The following external cohorts were used in the genetic analysis in the current study

1. Biobank Japan (BBJ)

The BBJ is a prospective cohort with over 200,000 patients with information on the diagnosis of 47 common diseases, enrolled from 12 cooperative medical institutes across Japan from June 2003 to March 2008. Baseline DNA, serum, and clinical information were collected, with annual follow-up for clinical data and survival data [1]. Data for genome-wide association analysis of common disease were available from a previous study [2].

2. UK Biobank (UKBB)

The UKBB is a prospective cohort study with over 500,000 participants from the general population, aged 37 to 73, enrolled between 2006 and 2010 from 22 assessment centers with baseline clinical information and biological samples, genotype, and linked healthcare record data [3]. Data for genome-wide association analysis of 660 binary traits and 118 non-binary traits were available from a previous study [4].

**References**

[1]	Nagai A, Hirata M, Kamatani Y, et al. (2017) Overview of the BioBank Japan Project: Study design and profile. J Epidemiol 27(3S): S2-S8. 10.1016/j.je.2016.12.005

[2]	Ishigaki K, Akiyama M, Kanai M, et al. (2020) Large-scale genome-wide association study in a Japanese population identifies novel susceptibility loci across different diseases. Nat Genet 52(7): 669-679. 10.1038/s41588-020-0640-3

[3]	Sudlow C, Gallacher J, Allen N, et al. (2015) UK biobank: an open access resource for identifying the causes of a wide range of complex diseases of middle and old age. PLoS Med 12(3): e1001779. 10.1371/journal.pmed.1001779

[4]	Canela-Xandri O, Rawlik K, Tenesa A (2018) An atlas of genetic associations in UK Biobank. Nat Genet 50(11): 1593-1599. 10.1038/s41588-018-0248-z

**Supplemental Table 1**. Associations between protein and incident acute myocardial infarction in SMART2D

|  |  | **Unadjusted** | | | **Adjusted** | | |
| --- | --- | --- | --- | --- | --- | --- | --- |
| **Protein** | **Long name** | **HR** | **95%CI** | **P** | **HR** | **95%CI** | **P** |
| NEFL | Neurofilament light polypeptide | 2.12 | 1.88-2.38 | 1.24E-36 | 1.70 | 1.43-2.01 | 6.52E-10 |
| EDA2R | Tumor necrosis factor receptor superfamily member 27 | 2.53 | 2.15-2.96 | 2.91E-30 | 2.25 | 1.69-2.97 | 1.41E-08 |
| CHRDL1 | Chordin-like protein 1 | 4.40 | 3.33-5.8 | 1.60E-25 | 2.69 | 1.83-3.94 | 4.35E-07 |
| FCER2 | Low affinity immunoglobulin epsilon Fc receptor | 2.31 | 1.9-2.78 | 5.18E-18 | 1.83 | 1.44-2.32 | 7.02E-07 |
| CXCL13 | C-X-C motif chemokine 13 | 1.56 | 1.33-1.82 | 1.70E-08 | 1.61 | 1.33-1.94 | 8.75E-07 |
| COL4A1 | Collagen alpha-1(IV) chain | 1.65 | 1.42-1.91 | 5.16E-11 | 1.59 | 1.31-1.92 | 1.65E-06 |
| RELT | Tumor necrosis factor receptor superfamily member 19L | 4.31 | 3.38-5.48 | 1.14E-32 | 3.25 | 1.99-5.29 | 2.08E-06 |
| TNFRSF10B | Tumor necrosis factor receptor superfamily member 10B | 3.87 | 3.04-4.91 | 1.47E-28 | 2.52 | 1.71-3.68 | 2.12E-06 |
| NTproBNP | N-terminal prohormone of brain natriuretic peptide | 1.46 | 1.33-1.6 | 5.01E-16 | 1.31 | 1.17-1.46 | 2.55E-06 |
| TNFRSF4 | Tumor necrosis factor receptor superfamily member 4 | 3.20 | 2.6-3.92 | 1.07E-28 | 2.04 | 1.5-2.74 | 3.47E-06 |
| FGF5 | Fibroblast growth factor 5 | 2.18 | 1.8-2.65 | 2.59E-15 | 1.91 | 1.44-2.53 | 6.53E-06 |
| COL18A1 | Collagen alpha-1(XVIII) chain | 9.43 | 6.23-14.27 | 2.61E-26 | 4.10 | 2.17-7.73 | 1.28E-05 |
| CCN5 | CCN family member 5 | 2.65 | 2.19-3.19 | 1.33E-24 | 1.81 | 1.38-2.36 | 1.36E-05 |
| JAM2 | Junctional adhesion molecule B | 2.76 | 2.31-3.29 | 4.23E-29 | 2.06 | 1.48-2.84 | 1.39E-05 |
| LAMA4 | Laminin subunit alpha-4 | 1.94 | 1.61-2.31 | 5.41E-13 | 1.76 | 1.35-2.28 | 1.88E-05 |
| CD74 | HLA class II histocompatibility antigen gamma chain | 3.71 | 2.86-4.81 | 3.31E-23 | 2.24 | 1.54-3.24 | 2.18E-05 |
| IGFBP4 | Insulin-like growth factor-binding protein 4 | 2.58 | 2.18-3.03 | 4.11E-30 | 2.10 | 1.48-2.95 | 2.42E-05 |

Hazard ratios (HR) with 95% confidence intervals (CI) are expressed per unit change in normalized protein expression. The adjusted model included age, sex, ethnicity, CVD history, smoking status, body mass index, diabetes duration, HbA1c, mean arterial pressure, low-density lipoprotein cholesterol, high-density lipoprotein cholesterol, triacylglycerol, usage of lipid-lowering medication, estimated glomerular filtration rate, and urine albumin-to-creatinine ratio as covariates. Only proteins with p < 0.05/1457=3.43x10^-5^ were shown, and proteins were sorted by ascending adjusted p.

**Supplemental Table 2.** Association between proteins and incident AMI accounting for non-AMI death as competing risk in SMART2D

|  |  | **Unadjusted** | | | **Adjusted** | | |
| --- | --- | --- | --- | --- | --- | --- | --- |
| **Protein** | **Long name** | **HR** | **95%CI** | **P** | **HR** | **95%CI** | **P** |
| EDA2R | Tumor necrosis factor receptor superfamily member 27 | 2.27 | 1.95-2.63 | 3.97E-27 | 2.04 | 1.55-2.67 | 2.45E-07 |
| FGF5 | Fibroblast growth factor 5 | 2.15 | 1.78-2.58 | 7.44E-16 | 1.91 | 1.49-2.43 | 2.57E-07 |
| CHRDL1 | Chordin-like protein 1 | 4.10 | 3.24-5.18 | 4.68E-32 | 2.64 | 1.82-3.82 | 2.73E-07 |
| NEFL | Neurofilament light polypeptide | 1.82 | 1.58-2.08 | 2.13E-17 | 1.52 | 1.28-1.79 | 8.56E-07 |
| FCER2 | Low affinity immunoglobulin epsilon Fc receptor | 2.18 | 1.81-2.63 | 2.02E-16 | 1.80 | 1.40-2.30 | 3.29E-06 |
| COL4A1 | Collagen alpha-1(IV) chain | 1.62 | 1.44-1.82 | 1.75E-15 | 1.54 | 1.26-1.86 | 1.10E-05 |
| CCN5 | CCN family member 5 | 2.57 | 2.14-3.07 | 4.97E-25 | 1.80 | 1.38-2.34 | 1.37E-05 |
| CXCL13 | C-X-C motif chemokine 13 | 1.51 | 1.31-1.74 | 1.27E-08 | 1.52 | 1.26-1.84 | 1.38E-05 |
| LAMA4 | Laminin subunit alpha-4 | 1.94 | 1.60-2.34 | 5.30E-12 | 1.74 | 1.34-2.27 | 3.52E-05 |
| COL18A1 | Collagen alpha-1(XVIII) chain | 7.94 | 5.3-11.87 | 6.55E-24 | 3.72 | 1.97-7.00 | 4.89E-05 |
| TNFRSF10B | Tumor necrosis factor receptor superfamily member 10B | 3.24 | 2.57-4.08 | 2.78E-23 | 2.16 | 1.48-3.13 | 5.99E-05 |
| JAM2 | Junctional adhesion molecule B | 2.66 | 2.12-3.33 | 1.73E-17 | 1.98 | 1.41-2.77 | 6.90E-05 |
| RELT | Tumor necrosis factor receptor superfamily member 19L | 3.58 | 2.84-4.50 | 2.18E-27 | 2.71 | 1.64-4.45 | 8.63E-05 |
| TNFRSF4 | Tumor necrosis factor receptor superfamily member 4 | 2.83 | 2.26-3.53 | 3.75E-20 | 1.82 | 1.34-2.47 | 1.27E-04 |
| NTproBNP | N-terminal prohormone of brain natriuretic peptide | 1.38 | 1.25-1.51 | 1.56E-11 | 1.23 | 1.09-1.38 | 3.71E-04 |
| CD74 | HLA class II histocompatibility antigen gamma chain | 3.37 | 2.61-4.33 | 6.09E-21 | 2.09 | 1.38-3.13 | 4.05E-04 |
| IGFBP4 | Insulin-like growth factor-binding protein 4 | 2.30 | 1.96-2.68 | 9.63E-26 | 1.84 | 1.29-2.62 | 6.77E-04 |

Hazard ratios (HR) with 95% confidence intervals (CI) are expressed per unit change in normalized protein expression. The adjusted model included age, sex, ethnicity, CVD history, smoking status, body mass index, diabetes duration, HbA1c, mean arterial pressure, low-density lipoprotein cholesterol, high-density lipoprotein cholesterol, triacylglycerol, usage of lipid-lowering medication, estimated glomerular filtration rate, and urine albumin-to-creatinine ratio as covariates. Only proteins identified from Supplemental Table 1 were shown, and proteins were sorted by ascending adjusted p-value.

**Supplemental Table 3.** Associations between protein and non-fatal incident AMI in SMART2D

|  |  |  | **Unadjusted** | | | **Adjusted** | | |
| --- | --- | --- | --- | --- | --- | --- | --- | --- |
| **Protein** | **Long name** | **Total AMI?** | **HR** | **95%CI** | **P** | **HR** | **95%CI** | **P** |
| NEFL | Neurofilament light polypeptide | Y | 2.11 | 1.87-2.38 | 4.98E-34 | 1.71 | 1.44-2.04 | 1.08E-09 |
| EDA2R | Tumor necrosis factor receptor superfamily member 27 | Y | 2.54 | 2.15-2.99 | 1.17E-28 | 2.33 | 1.74-3.10 | 1.06E-08 |
| FCER2 | Low affinity immunoglobulin epsilon Fc receptor | Y | 2.45 | 2.02-2.98 | 1.61E-19 | 1.98 | 1.55-2.54 | 5.84E-08 |
| CXCL13 | C-X-C motif chemokine 13 | Y | 1.60 | 1.37-1.88 | 3.72E-09 | 1.65 | 1.36-2.00 | 3.73E-07 |
| TNFRSF4 | Tumor necrosis factor receptor superfamily member 4 | Y | 3.29 | 2.66-4.05 | 1.03E-28 | 2.16 | 1.59-2.93 | 8.01E-07 |
| RELT | Tumor necrosis factor receptor superfamily member 19L | Y | 4.42 | 3.45-5.67 | 7.16E-32 | 3.52 | 2.13-5.82 | 9.41E-07 |
| FGF5 | Fibroblast growth factor 5 | Y | 2.22 | 1.82-2.70 | 3.36E-15 | 2.01 | 1.51-2.66 | 1.24E-06 |
| CHRDL1 | Chordin-like protein 1 | Y | 4.29 | 3.21-5.74 | 6.42E-23 | 2.61 | 1.74-3.91 | 3.17E-06 |
| TNFRSF8 | Tumor necrosis factor receptor superfamily member 8 | N | 2.57 | 2.08-3.18 | 1.97E-18 | 1.86 | 1.42-2.44 | 6.27E-06 |
| CD74 | HLA class II histocompatibility antigen gamma chain | Y | 3.87 | 2.97-5.06 | 2.88E-23 | 2.39 | 1.63-3.49 | 7.47E-06 |
| TNFRSF10B | Tumor necrosis factor receptor superfamily member 10B | Y | 3.82 | 2.99-4.90 | 1.87E-26 | 2.45 | 1.65-3.64 | 8.52E-06 |
| COL4A1 | Collagen alpha-1(IV) chain | Y | 1.63 | 1.39-1.90 | 1.68E-09 | 1.56 | 1.28-1.90 | 1.04E-05 |
| IGFBP4 | Insulin-like growth factor-binding protein 4 | Y | 2.62 | 2.22-3.10 | 1.90E-29 | 2.22 | 1.55-3.16 | 1.13E-05 |
| COL6A3 | Collagen alpha-3(VI) chain | N | 3.03 | 2.46-3.72 | 4.70E-26 | 2.21 | 1.55-3.16 | 1.31E-05 |
| PDCD1 | Programmed cell death protein 1 | N | 2.31 | 1.85-2.88 | 1.18E-13 | 1.86 | 1.40-2.46 | 1.47E-05 |
| NTproBNP | N-terminal prohormone of brain natriuretic peptide | Y | 1.44 | 1.31-1.59 | 6.65E-14 | 1.29 | 1.15-1.45 | 1.51E-05 |
| JAM2 | Junctional adhesion molecule B | Y | 2.78 | 2.31-3.33 | 6.54E-28 | 2.05 | 1.47-2.86 | 2.19E-05 |

Y(Yes) and N(No) indicate a significant association with total AMI in primary analysis including fatal and non-fatal AMI events. Hazard ratios (HR) with 95% confidence intervals (CI) are expressed per unit change in normalized protein expression. The adjusted model included age, sex, ethnicity, CVD history, smoking status, body mass index, diabetes duration, HbA1c, mean arterial pressure, low-density lipoprotein cholesterol, high-density lipoprotein cholesterol, triacylglycerol, usage of lipid-lowering medication, estimated glomerular filtration rate, and urine albumin-to-creatinine ratio as covariates. Only proteins identified from Supplemental Table 1 were shown, and proteins were sorted by ascending adjusted p-value.

**Supplemental Table 4.** Associations between proteins and incident AMI in T2D stratifying by sex

| **Protein** | **Long name** | **Male (n=921)** | | | | **Female (n=909)** | | | | **P-interaction** |
| --- | --- | --- | --- | --- | --- | --- | --- | --- | --- | --- |
|  |  | **HR** | **95%** | | **P** | **HR** | **95%** | | **P** |  |
| NTproBNP | N-terminal prohormone of brain natriuretic peptide | 1.30 | 1.12 | 1.50 | 4.66E-04 | 1.38 | 1.14 | 1.67 | 8.57E-04 | 0.069 |
| COL4A1 | Collagen alpha-1(IV) chain | 1.47 | 1.11 | 1.95 | 6.56E-03 | 1.76 | 1.34 | 2.30 | 3.62E-05 | 0.832 |
| COL18A1 | Collagen alpha-1(XVIII) chain | 3.80 | 1.74 | 8.30 | 7.87E-04 | 5.57 | 1.77 | 17.51 | 3.29E-03 | 0.458 |
| FGF5 | Fibroblast growth factor 5 | 2.61 | 1.63 | 4.18 | 7.06E-05 | 1.62 | 1.05 | 2.50 | 2.90E-02 | 0.036 |
| TNFRSF4 | Tumor necrosis factor receptor superfamily member 4 | 2.69 | 1.80 | 4.03 | 1.50E-06 | 1.35 | 0.80 | 2.28 | 2.63E-01 | 0.230 |
| LAMA4 | Laminin subunit alpha-4 | 1.59 | 1.13 | 2.23 | 7.70E-03 | 2.25 | 1.46 | 3.47 | 2.23E-04 | 0.355 |
| CHRDL1 | Chordin-like protein 1 | 2.53 | 1.59 | 4.00 | 8.02E-05 | 3.36 | 1.58 | 7.16 | 1.65E-03 | 0.937 |
| NEFL | Neurofilament light polypeptide | 1.61 | 1.29 | 2.00 | 2.43E-05 | 1.97 | 1.50 | 2.59 | 1.08E-06 | 0.255 |
| TNFRSF10B | Tumor necrosis factor receptor superfamily member 10B | 2.08 | 1.28 | 3.36 | 2.98E-03 | 3.78 | 1.95 | 7.32 | 7.97E-05 | 0.255 |
| CXCL13 | C-X-C motif chemokine 13 | 1.64 | 1.28 | 2.11 | 1.15E-04 | 1.60 | 1.18 | 2.17 | 2.61E-03 | 0.636 |
| CD74 | HLA class II histocompatibility antigen gamma chain | 2.41 | 1.54 | 3.77 | 1.24E-04 | 1.99 | 1.02 | 3.87 | 4.33E-02 | 0.642 |
| JAM2 | Junctional adhesion molecule B | 1.92 | 1.27 | 2.90 | 1.98E-03 | 2.43 | 1.39 | 4.24 | 1.80E-03 | 0.433 |
| CCN5 | CCN family member 5 | 1.76 | 1.28 | 2.42 | 4.95E-04 | 1.99 | 1.19 | 3.34 | 8.84E-03 | 0.625 |
| FCER2 | Low affinity immunoglobulin epsilon Fc receptor | 2.33 | 1.72 | 3.14 | 3.59E-08 | 1.24 | 0.82 | 1.87 | 3.04E-01 | 0.306 |
| RELT | Tumor necrosis factor receptor superfamily member 19L | 3.47 | 1.90 | 6.35 | 5.34E-05 | 3.20 | 1.32 | 7.76 | 1.01E-02 | 0.929 |
| IGFBP4 | Insulin-like growth factor-binding protein 4 | 2.07 | 1.35 | 3.16 | 7.98E-04 | 2.56 | 1.37 | 4.77 | 3.16E-03 | 0.445 |
| EDA2R | Tumor necrosis factor receptor superfamily member 27 | 2.39 | 1.71 | 3.36 | 4.11E-07 | 2.22 | 1.33 | 3.72 | 2.45E-03 | 0.444 |

Hazard ratios (HR) with 95% confidence intervals (CI) are expressed per unit change in normalized protein expression. The adjusted model included age, ethnicity, CVD history, smoking status, body mass index, diabetes duration, HbA1c, mean arterial pressure, low-density lipoprotein cholesterol, high-density lipoprotein cholesterol, triacylglycerol, usage of lipid-lowering medication, estimated glomerular filtration rate, and urine albumin-to-creatinine ratio as covariates.

**Supplemental Table 5.** Associations between proteins and incident AMI in T2D stratifying by baseline CVD history

|  |  | **Without CVD history (n=1713)** | | | | **With CVD history (n=116)** | | | | **P-interaction** |
| --- | --- | --- | --- | --- | --- | --- | --- | --- | --- | --- |
| **Protein** | **Long name** | **HR** | **95%** | | **P** | **HR** | **95%** | | **P** |  |
| NTproBNP | N-terminal prohormone of brain natriuretic peptide | 1.34 | 1.18 | 1.51 | 3.46E-06 | 1.45 | 0.96 | 2.19 | 7.57E-02 | 0.221 |
| COL4A1 | Collagen alpha-1(IV) chain | 1.54 | 1.25 | 1.89 | 3.67E-05 | 2.63 | 1.26 | 5.50 | 1.02E-02 | 0.187 |
| COL18A1 | Collagen alpha-1(XVIII) chain | 3.78 | 1.92 | 7.43 | 1.15E-04 | 13.11 | 0.55 | 312.70 | 1.12E-01 | 0.483 |
| FGF5 | Fibroblast growth factor 5 | 2.04 | 1.54 | 2.71 | 7.76E-07 | 0.73 | 0.21 | 2.55 | 6.17E-01 | 0.124 |
| TNFRSF4 | Tumor necrosis factor receptor superfamily member 4 | 1.99 | 1.43 | 2.78 | 4.98E-05 | 2.14 | 0.78 | 5.88 | 1.41E-01 | 0.365 |
| LAMA4 | Laminin subunit alpha-4 | 1.80 | 1.38 | 2.35 | 1.44E-05 | 1.46 | 0.23 | 9.25 | 6.86E-01 | 0.878 |
| CHRDL1 | Chordin-like protein 1 | 2.67 | 1.78 | 4.01 | 2.31E-06 | 3.92 | 0.49 | 31.64 | 2.00E-01 | 0.838 |
| NEFL | Neurofilament light polypeptide | 1.71 | 1.44 | 2.05 | 2.21E-09 | 1.42 | 0.74 | 2.73 | 2.96E-01 | 0.225 |
| TNFRSF10B | Tumor necrosis factor receptor superfamily member 10B | 2.35 | 1.54 | 3.59 | 8.04E-05 | 4.93 | 1.33 | 18.31 | 1.72E-02 | 0.528 |
| CXCL13 | C-X-C motif chemokine 13 | 1.55 | 1.27 | 1.90 | 1.90E-05 | 2.55 | 1.00 | 6.51 | 5.01E-02 | 0.272 |
| CD74 | HLA class II histocompatibility antigen gamma chain | 1.97 | 1.32 | 2.95 | 1.01E-03 | 5.13 | 1.62 | 16.21 | 5.36E-03 | 0.790 |
| JAM2 | Junctional adhesion molecule B | 2.10 | 1.50 | 2.94 | 1.68E-05 | 1.10 | 0.14 | 8.62 | 9.26E-01 | 0.413 |
| CCN5 | CCN family member 5 | 1.80 | 1.36 | 2.38 | 4.02E-05 | 1.99 | 0.52 | 7.67 | 3.17E-01 | 0.496 |
| FCER2 | Low affinity immunoglobulin epsilon Fc receptor | 1.79 | 1.38 | 2.33 | 1.40E-05 | 1.99 | 0.93 | 4.27 | 7.54E-02 | 0.271 |
| RELT | Tumor necrosis factor receptor superfamily member 19L | 3.20 | 1.89 | 5.40 | 1.39E-05 | 3.47 | 0.56 | 21.57 | 1.82E-01 | 0.124 |
| IGFBP4 | Insulin-like growth factor-binding protein 4 | 2.18 | 1.49 | 3.17 | 4.96E-05 | 1.47 | 0.46 | 4.64 | 5.13E-01 | 0.062 |
| EDA2R | Tumor necrosis factor receptor superfamily member 27 | 2.24 | 1.66 | 3.03 | 1.58E-07 | 2.22 | 0.81 | 6.08 | 1.22E-01 | 0.331 |

Hazard ratios (HR) with 95% confidence intervals (CI) are expressed per unit change in normalized protein expression. The adjusted model included age, ethnicity, CVD history, smoking status, body mass index, diabetes duration, HbA1c, mean arterial pressure, low-density lipoprotein cholesterol, high-density lipoprotein cholesterol, triacylglycerol, usage of lipid-lowering medication, estimated glomerular filtration rate, and urine albumin-to-creatinine ratio as covariates.

**Supplemental Table 6.** Associations between proteins and incident AMI in T2D stratifying by baseline DKD history

| **Protein** | **Long name** | **Without DKD (n=959)** | | | | **With DKD (n=861)** | | | | **P-interaction** |
| --- | --- | --- | --- | --- | --- | --- | --- | --- | --- | --- |
|  |  | **HR** | **95%** | | **P** | **HR** | **95%** | | **P** |  |
| NTproBNP | N-terminal prohormone of brain natriuretic peptide | 1.26 | 0.99 | 1.61 | 6.29E-02 | 1.36 | 1.19 | 1.55 | 4.93E-06 | 0.659 |
| COL4A1 | Collagen alpha-1(IV) chain | 1.04 | 0.66 | 1.64 | 8.70E-01 | 1.70 | 1.39 | 2.08 | 3.44E-07 | 0.330 |
| COL18A1 | Collagen alpha-1(XVIII) chain | 2.16 | 0.64 | 7.32 | 2.14E-01 | 4.38 | 2.03 | 9.47 | 1.75E-04 | 0.692 |
| FGF5 | Fibroblast growth factor 5 | 2.23 | 1.38 | 3.61 | 1.01E-03 | 1.72 | 1.22 | 2.43 | 2.21E-03 | 0.492 |
| TNFRSF4 | Tumor necrosis factor receptor superfamily member 4 | 1.93 | 1.14 | 3.26 | 1.49E-02 | 2.19 | 1.47 | 3.25 | 1.08E-04 | 0.553 |
| LAMA4 | Laminin subunit alpha-4 | 1.28 | 0.68 | 2.44 | 4.45E-01 | 1.84 | 1.36 | 2.48 | 7.66E-05 | 0.340 |
| CHRDL1 | Chordin-like protein 1 | 2.19 | 0.94 | 5.07 | 6.83E-02 | 2.78 | 1.78 | 4.36 | 7.81E-06 | 0.676 |
| NEFL | Neurofilament light polypeptide | 2.04 | 1.49 | 2.80 | 9.50E-06 | 1.54 | 1.25 | 1.89 | 4.96E-05 | 0.007 |
| TNFRSF10B | Tumor necrosis factor receptor superfamily member 10B | 2.46 | 1.17 | 5.18 | 1.80E-02 | 2.47 | 1.56 | 3.92 | 1.21E-04 | 0.189 |
| CXCL13 | C-X-C motif chemokine 13 | 1.47 | 1.01 | 2.12 | 4.26E-02 | 1.67 | 1.34 | 2.10 | 7.31E-06 | 0.396 |
| CD74 | HLA class II histocompatibility antigen gamma chain | 2.21 | 1.18 | 4.13 | 1.30E-02 | 2.05 | 1.30 | 3.24 | 2.14E-03 | 0.692 |
| JAM2 | Junctional adhesion molecule B | 1.53 | 0.78 | 3.00 | 2.12E-01 | 2.31 | 1.52 | 3.51 | 9.56E-05 | 0.382 |
| CCN5 | CCN family member 5 | 1.35 | 0.73 | 2.50 | 3.47E-01 | 1.84 | 1.36 | 2.50 | 8.01E-05 | 0.529 |
| FCER2 | Low affinity immunoglobulin epsilon Fc receptor | 2.29 | 1.44 | 3.65 | 4.65E-04 | 1.67 | 1.24 | 2.24 | 8.15E-04 | 0.288 |
| RELT | Tumor necrosis factor receptor superfamily member 19L | 4.16 | 1.67 | 10.37 | 2.21E-03 | 3.11 | 1.71 | 5.65 | 2.05E-04 | 0.073 |
| IGFBP4 | Insulin-like growth factor-binding protein 4 | 2.27 | 1.19 | 4.32 | 1.30E-02 | 1.88 | 1.24 | 2.85 | 2.85E-03 | 0.031 |
| EDA2R | Tumor necrosis factor receptor superfamily member 27 | 2.33 | 1.29 | 4.20 | 4.80E-03 | 2.20 | 1.58 | 3.05 | 2.35E-06 | 0.048 |

Hazard ratios (HR) with 95% confidence intervals (CI) are expressed per unit change in normalized protein expression. The adjusted model included age, ethnicity, CVD history, smoking status, body mass index, diabetes duration, HbA1c, mean arterial pressure, low-density lipoprotein cholesterol, high-density lipoprotein cholesterol, triacylglycerol, usage of lipid-lowering medication, estimated glomerular filtration rate, and urine albumin-to-creatinine ratio as covariates.

**Supplemental Table 7.** Associations between proteins and incident AMI in T2D stratifying by age groups

| **Protein** | **Long name** | **Age group (tertile1: 21 to 53 years) (n=616)** | | | | **Age group (tertile2: 54 to 62 years) (n=662)** | | | | **Age group (tertile3: 63 to 89 years) (n=552)** | | | | **P-interaction** | |
| --- | --- | --- | --- | --- | --- | --- | --- | --- | --- | --- | --- | --- | --- | --- | --- |
|  |  | **HR** | **95%** | | **P** | **HR** | **95%** | | **P** | **HR** | **95%** | | **P** | **P2** | **P3** |
| NTproBNP | N-terminal prohormone of brain natriuretic peptide | 0.99 | 0.76 | 1.29 | 9.33E-01 | 1.32 | 1.08 | 1.60 | 5.99E-03 | 1.57 | 1.28 | 1.91 | 1.31E-05 | 0.065 | 0.186 |
| COL4A1 | Collagen alpha-1(IV) chain | 1.31 | 0.92 | 1.88 | 1.32E-01 | 1.42 | 0.99 | 2.04 | 5.79E-02 | 1.99 | 1.46 | 2.71 | 1.17E-05 | 0.946 | 0.335 |
| COL18A1 | Collagen alpha-1(XVIII) chain | 2.20 | 0.78 | 6.23 | 1.38E-01 | 1.35 | 0.40 | 4.53 | 6.29E-01 | 24.82 | 7.23 | 85.21 | 3.33E-07 | 0.209 | 0.446 |
| FGF5 | Fibroblast growth factor 5 | 2.41 | 1.58 | 3.68 | 4.64E-05 | 2.04 | 1.10 | 3.77 | 2.36E-02 | 1.87 | 1.01 | 3.44 | 4.51E-02 | 0.583 | 0.772 |
| TNFRSF4 | Tumor necrosis factor receptor superfamily member 4 | 2.29 | 1.19 | 4.38 | 1.28E-02 | 1.35 | 0.79 | 2.30 | 2.69E-01 | 2.77 | 1.61 | 4.74 | 2.12E-04 | 0.038 | 0.349 |
| LAMA4 | Laminin subunit alpha-4 | 1.43 | 0.95 | 2.16 | 8.79E-02 | 1.38 | 0.73 | 2.59 | 3.20E-01 | 3.33 | 2.01 | 5.53 | 3.06E-06 | 0.891 | 0.037 |
| CHRDL1 | Chordin-like protein 1 | 1.63 | 0.85 | 3.13 | 1.43E-01 | 2.37 | 1.03 | 5.46 | 4.19E-02 | 5.77 | 2.77 | 12.00 | 2.71E-06 | 0.903 | 0.632 |
| NEFL | Neurofilament light polypeptide | 1.58 | 1.14 | 2.21 | 6.69E-03 | 1.81 | 1.35 | 2.45 | 9.55E-05 | 1.90 | 1.42 | 2.55 | 1.87E-05 | 0.979 | 0.480 |
| TNFRSF10B | Tumor necrosis factor receptor superfamily member 10B | 1.45 | 0.66 | 3.20 | 3.58E-01 | 1.61 | 0.84 | 3.07 | 1.49E-01 | 6.28 | 3.17 | 12.47 | 1.48E-07 | 0.849 | 0.499 |
| CXCL13 | C-X-C motif chemokine 13 | 1.50 | 1.06 | 2.12 | 2.04E-02 | 1.51 | 1.08 | 2.11 | 1.59E-02 | 1.92 | 1.37 | 2.68 | 1.50E-04 | 0.758 | 0.161 |
| CD74 | HLA class II histocompatibility antigen gamma chain | 1.59 | 0.84 | 3.03 | 1.55E-01 | 1.35 | 0.67 | 2.70 | 4.00E-01 | 3.91 | 2.06 | 7.42 | 3.18E-05 | 0.848 | 0.534 |
| JAM2 | Junctional adhesion molecule B | 1.84 | 1.14 | 2.95 | 1.19E-02 | 1.47 | 0.67 | 3.21 | 3.32E-01 | 4.00 | 1.67 | 9.58 | 1.86E-03 | 0.918 | 0.171 |
| CCN5 | CCN family member 5 | 1.36 | 0.91 | 2.04 | 1.36E-01 | 1.89 | 1.09 | 3.28 | 2.40E-02 | 2.79 | 1.62 | 4.82 | 2.20E-04 | 0.691 | 0.474 |
| FCER2 | Low affinity immunoglobulin epsilon Fc receptor | 2.39 | 1.46 | 3.90 | 5.15E-04 | 1.57 | 1.03 | 2.40 | 3.40E-02 | 1.77 | 1.20 | 2.59 | 3.56E-03 | 0.566 | 0.335 |
| RELT | Tumor necrosis factor receptor superfamily member 19L | 1.64 | 0.64 | 4.16 | 3.00E-01 | 2.35 | 0.98 | 5.65 | 5.70E-02 | 6.50 | 2.79 | 15.18 | 1.49E-05 | 0.525 | 0.333 |
| IGFBP4 | Insulin-like growth factor-binding protein 4 | 1.67 | 0.85 | 3.28 | 1.35E-01 | 1.17 | 0.64 | 2.15 | 6.03E-01 | 4.53 | 2.37 | 8.65 | 4.68E-06 | 0.320 | 0.586 |
| EDA2R | Tumor necrosis factor receptor superfamily member 27 | 2.57 | 1.49 | 4.45 | 7.11E-04 | 1.59 | 0.96 | 2.64 | 6.90E-02 | 2.89 | 1.77 | 4.72 | 2.16E-05 | 0.474 | 0.391 |

Hazard ratios (HR) with 95% confidence intervals (CI) are expressed per unit change in normalized protein expression. The adjusted model included age, ethnicity, CVD history, smoking status, body mass index, diabetes duration, HbA1c, mean arterial pressure, low-density lipoprotein cholesterol, high-density lipoprotein cholesterol, triacylglycerol, usage of lipid-lowering medication, estimated glomerular filtration rate, and urine albumin-to-creatinine ratio as covariates. P2 refers to the p-value for interaction between age tertile 2 and protein, P3 refers to the p-value for interaction between age tertile 3 and protein

**Supplemental Table 8.** Associations between 17 proteins identified in primary analysis and incident AMI the UKBB

| **Group** | **Protein** | **HR (95% CI)** | **P** | **Adj P** |
| --- | --- | --- | --- | --- |
| 1 | LAMA4 | 1.07 (1.03,1.12) | 2.88E-04 | 8.37E-01 |
| 1 | NTproBNP | 1.48 (1.43, 1.53) | 4.30E-117 | 1.25E-113 |
| 1 | NEFL | 1.14 (1.09,1.19) | 2.55E-09 | 7.42E-06 |
| 1 | FGF5 | 1.07 (1.03,1.11) | 1.17E-03 | 1.00E-00 |
| 1 | FCER2 | 1.09 (1.05, 1.13) | 3.71E-06 | 1.08E-02 |
|  |  |  |  |  |
| 2 | EDA2R | 1.30 (1.24,1.35) | 1.33E-33 | 3.88E-30 |
| 2 | CHRDL1 | 1.15 (1.10, 1.20) | 2.71E-11 | 7.88E-08 |
| 2 | CXCL13 | 1.14 (1.11,1.18) | 9.93E-19 | 2.89E-15 |
| 2 | COL4A1 | 1.08 (1.03,1.12) | 2.48E-04 | 7.22E-01 |
| 2 | RELT | 1.23 (1.19, 1.28) | 9.60E-27 | 2.79E-23 |
| 2 | TNFRSF10B | 1.13 (1.11, 1.16) | 3.85E-24 | 1.12E-20 |
| 2 | COL18A1 | 1.27 (1.22,1.32) | 2.56E-32 | 7.47E-29 |
| 2 | CCN5 | 1.14 (1.09,1.19) | 1.80E-09 | 5.25E-06 |
| 2 | JAM2 | 1.09 (1.05,1.13) | 5.03E-06 | 1.46E-02 |
| 2 | CD74 | 1.21 (1.16,1.25) | 5.88E-21 | 1.71E-17 |
| 2 | IGFBP4 | 1.22 (1.18,1.27) | 1.79E-26 | 5.22E-23 |

Hazard ratios (HR) with 95% confidence intervals (CI) show the strength of associations adjusted for age, sex, race, deprivation index, education, smoking, alcohol drinking, physical activity, dietary quality, body mass index, systolic blood pressure, and diabetes as reported by Ho FK et al (PMID: 39540306). P values were corrected for multiple testing using the Bonferroni procedure in the initial report (Adj P). Group 1 indicates proteins independently associated with incident AMI in the SMART2D cohort and improved AMI risk prediction. Group 2 indicates remaining proteins identified to be associated with incident AMI in SMART2D in primary analysis.

**Supplemental Table 9:** Literature summary of 17 proteins associated with incident AMI in SMART2D cohort

| **Proteins** | **Associated with AMI** | **Study population** | **Associated with AMI in T2D** | **Main biological function** | **Causality supported by MR or experimental studies** | **Related to other CVD outcome?** | **Reference (PMID)** | **Correlation within SMART2D (P<0.0001)** |
| --- | --- | --- | --- | --- | --- | --- | --- | --- |
| CCN5 | Elevated CCN5 levels associated with increased risk of incident AMI [1]. | UKB | - | Matricellular protein; inhibits vascular smooth muscle cell proliferation and fibrosis [2]. | In vitro studies showed CCN5 inhibits cellular senescence in cardiac myoblasts and fibroblasts [3]. CCN5 knockout mice exhibit lipotoxic cardiomyopathy with mild obesity and diabetes [4] | Associated with HF, Stroke, MACE in UKB [1]. | [1] 39540306 [2] 27150688 [3] 41721008 [4] 30485307 | Age (+), BMI (+), diabetes duration (+), MAP (+), TG (+), HDL (-) |
| CD74 | Elevated CD74 levels associated with increased risk of incident AMI and CHD [1,2]. | UKB/CKB | - | MHC class II chaperone; cell surface receptor for MIF and MIF-2; regulates macrophage inflammation, T- and B-cell development, and dendritic cell motility [3]. | High expression of CD74 in mouse model of myocardial infarction compared to controls, suggesting potential biomarker of MI [4]. Animal models revealed that CD74 ablation protects against T2D-induced cardiac remodelling [5]. | Associated with HF, Stroke, MACE in UKB [1]. | [1] 39540306 [2] 39322770 [3] 27752708 [4] 40746991 [5] 35033649 | Age (+), BMI (+), diabetes duration (+), Hba1c (+), MAP (+), TG (+), HDL (-) |
| CHRDL1 | Elevated CHRDL1 levels associated with increased risk of incident AMI and CHD [1,2]. | UKB/CKB | - | CHRDL1 is a bone morphogenetic protein antagonist involved in cell development and differentiation [3]. | - | Associated with HF, Stroke, MACE in UKB [1]. | [1] 39540306 [2] 39322770 [3] 36682027 | Age (+), diabetes duration (+), MAP (+), TG (+) |
| COL18A1 | Elevated COL18A1 levels associated with increased risk of incident AMI and CHD [1,2]. | UKB/CKB | - | COL18A1 is a basement membrane protein with anti-angiogenic properties [3] | - | Associated with HF, Stroke, MACE in UKB [1]. | [1] 39540306 [2] 39322770 [3] 15313955 | Age (+), diabetes duration (+), MAP (+), TG (+), HDL (-) |
| COL4A1 | - | - | - | COL4A1 is a major component of basement membranes, maintaining vascular and tissue integrity [2] | Studies in animal models showed that COL4A1 mutations caused cardiac defects [3]. | Associated with HF and Stroke in UKB [1]. | [1] 39540306 [2] 20166936  [3] 20056676 | Age (+), BMI (-), diabetes duration (+) |
| CXCL13 | Elevated CXCL13 levels associated with increased risk of incident AMI [1] and CAD [2] | UKB | - | CXCL13 plays a multifaceted role in CVD, primarily acting as a potent B-cell chemoattractant that modulates inflammation, immune response, and tissue remodelling [3]. | Knockdown of CXCL13 in animal model improved vascular remodelling, reduced blood pressure, and protected the heart [4].MR analysis suggest causal role in CAD risk [2]. | Associated with HF, Stroke, MACE in UKB [1]. | [1] 39540306 [2] 42045057 [3] 33161233 [4] 41066312 | BMI (+), TG (+), LDL (+), HDL (-) |
| EDA2R | Elevated EDA2R levels associated with increased risk of incident AMI and CHD [1,2]. | UKB/CKB | - | EDA2R is a tissue ‑ independent aging marker linked to inflammation and age ‑related disorders [3]. | Knockdown of EDA2R exerted anti-apoptotic and antioxidant effects against Ischemia/reperfusion injury in vivo and in vitro by suppressing the NF-κB signalling pathway [4]. | Associated with HF, Stroke, MACE in UKB [1]. | [1] 39540306 [2] 39322770 [3] 39988718 [4] 38797667 | Age (+), diabetes duration (+), MAP (+), LDL (-) |
| FCER2 | Elevated FCER2 levels associated with increased risk of incident AMI [1]. | UKB | - | Low-affinity receptor for immunoglobulin E (IgE) and CR2/CD21. Has essential roles in the regulation of IgE production and in the differentiation of B cells [2]. | - | Associated with HF, Stroke, MACE in UKB [1]. | [1] 39540306 [2] 2167225 | Age (+), BMI (+), diabetes duration (+), MAP (+), TG (+), HDL (-) |
| FGF5 | - | - | - | FGF5 plays an important role in the regulation of cell proliferation and cell differentiation [1]. | FGF5 casually linked to CHD [2]. It also mediates effect of BP on heart failure susceptivity [3]. | Associated with HF and Stroke in UKB [4]. | [1] 33536494 [2] 39322770 [3] 38487880 [4] 39540306 | Age (+), BMI (-), diabetes duration (+), HDL (+) |
| IGFBP4 | Elevated IGFBP4 levels associated with increased risk of incident AMI and CHD [1,2]. | UKB/CKB | - | IGFBP4 regulates insulin-like growth factor activity, with effects on glucose metabolism and vascular function [3]. | MR analysis suggests a causal association between IGFBP4 and risk of myocardial infarction and ischemic stroke [2]. | Associated with HF, Stroke, MACE in UKB [1]. | [1] 39540306 [2] 39322770 [3] 29864720 | Age (+), BMI (+), diabetes duration (+), MAP (+), TG (+), HDL (-) |
| JAM2 | Elevated JAM2 levels associated with increased risk of incident AMI [1]. | UKB | - | JAM2 is involved in multiple functions, such as cell–cell contacts, the formation of vascular tubes, the homeostasis of stem cell niches, and the promotion of leukocyte transmigration during inflammation [2]. | - | Associated with HF, Stroke, MACE in UKB [1]. | [1] 39540306 [2] 39838844 | Age (+), BMI (+), diabetes duration (+), MAP (+), TG (+), HDL (-) |
| LAMA4 | - | - | - | LAMA4 plays a significant role in mediating cell adhesion and migration, as well as organizing cells into tissues during embryonic development by interacting with other extracellular matrix (ECM) components [1]. | - | Associated with HF and Stroke in UKB [2]. | [1] 34414238 [2] 39540306 | BMI (+), diabetes duration (+), TG (+), HDL (-) |
| NEFL | Elevated NEFL levels associated with increased risk of incident AMI and CHD [1,2]. | UKB/CKB | - | NEFL is a biomarker of axonal injury, reflecting damage to neuronal integrity. recent research indicates that blood levels are strongly associated with CVD risk [3]. | - | Associated with HF, Stroke, MACE in UKB [1]. | [1] 39540306 [2] 39322770 [3] 22319610 | Age (+), BMI (-), diabetes duration (+), HbA1c (+), MAP (+) |
| NTproBNP | Elevated NTproBNP levels associated with increased risk of incident AMI and CHD [1,2]. | UKB/CKB | - | NTproBNP is a cardiac stress biomarker released in response to ventricular wall stress [3]. | - | Associated with HF, Stroke, MACE in UKB [1]. | [1] 39540306 [2] 39322770 [3] 41247830 | Age (+), diabetes duration (+), MAP (+), LDL (-) |
| RELT | Elevated RELT levels associated with increased risk of incident AMI and CHD [1,2]. | UKB/CKB | - | RELT is a TNF receptor superfamily member involved in inflammation and cell death [3]. | - | Associated with HF, Stroke, MACE in UKB [1]. | [1] 39540306 [2] 39322770 [3] 31988141 | Age (+), BMI (+), diabetes duration (+), MAP (+), TG (+), HDL (-) |
| TNFRSF10B | Elevated TNFRSF10B levels associated with increased risk of incident AMI and CHD [1,2]. | UKB/CKB | - | TNFRSF10B is a pro-apoptotic receptor that mediates cell death and inflammation [3]. | - | Associated with HF, Stroke, MACE in UKB [1]. | [1] 39540306 [2] 39322770 [3] 32109381 | Age (+), BMI (+), diabetes duration (+), MAP (+), TG (+), HDL (-) |
| TNFRSF4 | Elevated TNFRSF4 levels associated with increased risk of incident AMI [1,2]. | UKB/CKB | - | TNFRSF4 is a T-cell co-stimulatory receptor involved in immune regulation [3]. | - | Associated with HF, Stroke, MACE in UKB [1]. | [1] 39540306 [2] 39322770 [3] 22956587 | Age (+), BMI (+), diabetes duration (+), HbA1c (+), MAP (+), TG (+), HDL (-) |

Abbreviations: UKB, UK Biobank; CKB, China Kadoorie Biobank.

**Supplemental Figure 1. Overview of study design**

**Supplemental Figure 2.** Functional and pathway enrichment analysis. Pathways that were significantly overrepresented by proteins associated with AMI. KEGG (Kyoto Encyclopedia of Genes and Genomes); CC, cellular component; MF, molecular function.

**Supplemental Figure 3.** Correlation among 17 proteins associated with incident AMI.

**Supplemental Figure 4.** Correlation between 17 proteins and metabolic risk factors. *p<0.01, **p<0.001, ***p<0.0001

**Supplemental Figure 5**: Harell’s C statistic for predicting incident AMI. The protein model includes LAMA4, NT-proBNP, NEFL, FGF5, FCER2, COL4A1, CXCL13 and EDA2R. Clinical variables include age, sex, ethnicity, CVD history, smoking status, body mass index, diabetes duration, HbA1c, mean arterial pressure, HDL-cholesterol, LDL-cholesterol, triacylglycerol, usage of lipid-lowering medications, baseline eGFR, and log-transformed uACR.

**Supplemental Figure 6.** Manhattan plot of genome wide association study of circulating NT-proBNP levels.

**Supplemental Figure 7.** Manhattan plot of genome wide association study of circulating FGF5 levels.

**Supplemental Figure 8.** Manhattan plot of genome wide association study of circulating FCER2 levels.
